# Bluetongue: a historical and epidemiological perspective with the emphasis on South Africa

**DOI:** 10.1186/1743-422X-9-198

**Published:** 2012-09-13

**Authors:** Peter Coetzee, Maria Stokstad, Estelle H Venter, Mette Myrmel, Moritz Van Vuuren

**Affiliations:** 1 Department of Veterinary Tropical Diseases, Faculty of Veterinary Medicine, University of Pretoria, Private Bag X04, Onderstepoort, Pretoria 0110, South Africa; 2 Department of Food Safety and Infection Biology, Norwegian School of Veterinary Science, P. O. Box 8146, Oslo, Norway; 3 Department of Production Animal Clinical Sciences, Norwegian School of Veterinary Science, P. O. Box 8146, 0033 Oslo, Norway

**Keywords:** Bluetongue virus, *Culicoides*, Serotype, Survey, African carnivores, African herbivores, Sheep, Cattle, Onderstepoort, South Africa, Control, Vaccine

## Abstract

Bluetongue (BT) is a non-contagious, infectious, arthropod transmitted viral disease of domestic and wild ruminants that is caused by the bluetongue virus (BTV), the prototype member of the *Orbivirus* genus in the family *Reoviridae*. Bluetongue was first described in South Africa, where it has probably been endemic in wild ruminants since antiquity. Since its discovery BT has had a major impact on sheep breeders in the country and has therefore been a key focus of research at the Onderstepoort Veterinary Research Institute in Pretoria, South Africa. Several key discoveries were made at this Institute, including the demonstration that the aetiological agent of BT was a dsRNA virus that is transmitted by *Culicoides* midges and that multiple BTV serotypes circulate in nature. It is currently recognized that BT is endemic throughout most of South Africa and 22 of the 26 known serotypes have been detected in the region. Multiple serotypes circulate each vector season with the occurrence of different serotypes depending largely on herd-immunity. Indigenous sheep breeds, cattle and wild ruminants are frequently infected but rarely demonstrate clinical signs, whereas improved European sheep breeds are most susceptible. The immunization of susceptible sheep remains the most effective and practical control measure against BT. In order to protect sheep against multiple circulating serotypes, three pentavalent attenuated vaccines have been developed. Despite the proven efficacy of these vaccines in protecting sheep against the disease, several disadvantages are associated with their use in the field.

## Introduction

Bluetongue (BT) is a non-contagious, infectious, arthropod transmitted viral disease of ruminant and camelid species which is caused by the bluetongue virus (BTV), the prototype member of the *Orbivirus* genus in the family *Reoviridae*[1]. Bluetongue virus contains ten linear dsRNA genome segments [2], that encode seven structural (VP1-VP7) and 5 non-structural proteins (NS1, NS2, NS3, NS3/A, NS4) [3,4]. Variation in the outer capsid proteins, especially VP2, determines serotype specificity [5] and 26 serotypes of the virus have been identified [6]. These serotypes exist in a complex network of serological cross relationships, varying from partial to no protection between heterologous strains [7]. Except for serotype diversity, the localized circulation of the virus in different ecosystems throughout the world has also led to the evolution of distinct geographical variants or topotypes of the virus. Bluetongue viruses are broadly divided into western and eastern lineages and further regional topotypes, based on phylogenetic analysis of nucleotide sequences from the majority of the genome segments. Viruses from the western lineages circulate in Africa, the Caribbean and the Americas, whereas those from eastern lineages are endemic in Asia, Indonesia and Australia [8,9]. The classification of BTV is complicated by the virus’s ability to reassort and/or recombine its genome segments in host or vector cells that have concurrently been infected with more than one strain or serotype of the virus [10-13]. It is possible that novel strains may arise in nature of which the genome segments (or portions of the genome segments) are not all derived from one parental virus [13-15]. It is therefore desirable that viral topotyping be conducted through the analysis of sequence data from more than a single genome segment.

Bluetongue virus is transmitted almost exclusively by adult female haematophagous midges that belong to the *Culicoides* genus [16]. Over 1400 species have been described, but only 30 or so have been implicated in the transmission of the virus [17]. The distribution of BT is closely linked to the distribution of vector competent midge species and climatic conditions that support a large population of the insects. Bluetongue is therefore endemic primarily in the tropical and sub-tropical regions between the latitudes of 40 °N and 35 °S, although in certain regions of North America and China the disease has been detected up to 50 °N [18]. Since 1998 there has been a dramatic change in the distribution of BT, with the disease having spread into countries of north-western Europe and Scandinavia [19,20]. There is a perception that the northward expansion and persistence of bluetongue in regions of Europe that were previously thought to be beyond the northernmost latitude where climatic conditions could sustain bluetongue, is partially attributable to the effects of global climate change [21]. Furthermore novel Palaeartic vectors (particularly the *obsoletus* and *pulicaris* species complexes) were involved in the transmission of bluetongue in northern Europe [17]. Due to the wide distribution of these midge species in the Palearctic region, the whole of Europe is considered to be continued risk of BT outbreaks and it can be expected that the introduction of new BTV strains and serotypes will continue to occur on a regular basis [22].

Bluetongue virus can infect most ruminant and camelid species but severe disease is usually only seen erratically in certain breeds of sheep (especially in European fine wool and mutton breeds) and some species of wild ruminants such as North American white tailed deer [23,24]. Cattle and goats are usually sub-clinically affected [23], although acute infections may occur when the virus spreads into immunologically naive populations in regions where it is not normally encountered. For example during the recent outbreak of BTV-8 in Europe, clinical disease was noted in both cattle and goats. Clinical signs documented in cattle included ocular discharge, conjunctivitis, oral mucosal congestion, development of ulcers and necrotic lesions on the lips and tongue as well as oedema [25], whereas goats demonstrated an acute drop in milk production, oedema of the lips and head, nasal discharge and erythema of the skin and udder [26]. Nevertheless in endemic regions cattle (and goats) are commonly considered to be amplifying hosts of BTV, due to a prolonged viraemia that usually occurs in the absence of clinical signs [27]. Other routes of transmission of BTV include venereal transmission through virus contaminated semen during the period of infectious viraemia [28] as well as direct contact transmission through the oral ingestion of contaminated placental or foetal material [29]. Infection of pregnant sheep and cattle with certain strains of the virus, especially attenuated vaccine strains (MLVs), may also lead to transplacental infection. Depending on the time of gestation when the foetus is infected, transplacental infection may result in either still births, abortions, or the birth of non-viable offspring with severe central nervous system deformities [30,31]. Notably the BTV-8 strain that recently spread throughout much of Europe demonstrated the capability to cross the placenta of ruminants to cause foetal infections at a high frequency, a property that had not previously been generally associated with a wild type strains of the virus [29,32,33]

Bluetongue virus preferentially infects endothelial cells lining the walls of blood vessels, as well mononuclear phagocytic and dendritic cells [34-36]. Virus-mediated damage to endothelial cells results in vascular thrombosis, tissue infarction, necrosis and haemorrhage [23,35,37,38]. These lesions manifest clinically in sheep as fever, serous to bloody nasal discharge, severe pulmonary and facial oedema, oral erosions and ulcers, lameness with hyperaemia of the coronary band and torticollis [23,38]. The clinical presentation of BT can also vary widely amongst susceptible sheep and can range from sub-clinical infections in the majority of cases to severe disease and death of infected animals. The mortality among susceptible sheep ranges from 2-30% [38] but can occasionally be as high as 70% [39]. Animals that recover from the disease typically demonstrate a protracted recovery period, during which they may show wasting and a reduction in fertility, milk production and the quality of wool [23,38].

The economic losses that are associated with BT can be substantial. It is estimated for example that worldwide economic losses associated with BT in 1996 exceeded 3 billion US dollars [40]. Except for direct economic losses that are caused by high morbidity and mortality, BT also causes significant losses due to imposition of trade restrictions on ruminants and their germ plasm from BT affected regions [41]. Other indirect losses include the costs that are associated with surveillance and mass vaccination, vector control and the treatment of clinically affected animals [42].

### History of bluetongue research in South Africa

Bluetongue has been known in Africa for over a hundred years and has probably been endemic in wild ruminants in sub-Saharan Africa since antiquity [38].The first official report of BT emanated from the Cape Province of South Africa in the late eighteenth century, following the importation of susceptible Merino sheep from Europe. The disease was initially called “fever” or “epizootic catarrh”, but was later referred to as “malarial catarrhal fever of sheep”, due to the mistaken belief that the disease was caused by an intracorpuscular parasite [43,44]. The disease was later renamed “bluetongue” (1905), with reference to the characteristic cyanotic tongues that were occasionally observed in infected sheep [45].

Bluetongue has been an important disease of sheep in southern Africa ever since its discovery and has therefore been a major focus of research at the Onderstepoort Veterinary Institute (OVI) in Pretoria, South Africa. Several key discoveries with regards to the nature of the aetiological agent and its epidemiology were made at the Institute [46]. In 1905, Sir Arnold Theiler demonstrated that the aetiological agent of BT could be passed through a filter, thus indicating that the disease was caused by a virus [47]. Theiler also introduced the first vaccine against BT that consisted of a single virus strain (BTV-4) that was attenuated by serial passage in sheep. This crude blood vaccine induced remarkable cross-protection to other serotypes and was used by sheep farmers in South Africa for over 40 years [48,49].

Based on circumstantial evidence such as a strong seasonal incidence and the fact that sheep could be protected by stabling at night and by avoiding low lying pastures, it was surmised that an insect vector was responsible for the transmission of BTV. The development of light traps at the OVI for capture of large numbers of *Culicoides* at night enabled Du Toit (1944) to demonstrate that BT could be induced in susceptible sheep that were inoculated with suspensions of wild caught *Culicoides imicola*. Du Toit subsequently confirmed that *Culicoides* spp. were biological vectors of BTV, when he demonstrated that *C. imicola* which had been fed on BTV infected sheep, could transmit the disease to healthy sheep after an extrinsic incubation period of several days in the insects [16].

Frequent failures of the vaccine that was prepared from the Theiler strain led Neitz in 1948 to investigate the plurality of BTV strains in nature by cross-protection studies in sheep. This work confirmed that sheep that had been infected with a particular strain developed lifelong protective immunity against re-infection with the same strain, but only partial to no protection against infection with heterologous field strains [50]. These findings led to the first studies on the serotyping of BTV field isolates, an essential step that was instrumental in the development of effective cross-protective vaccines against multiple circulating BTV serotypes. Howell and colleagues developed an *in vitro* serum virus neutralization test in the 1960s that they used to classify 22 southern African field isolates into 12 serotypes [51], a number which was expanded by an additional 4 serotypes by plaque inhibition assay in the 1970s [52]. The development of serotyping assays placed the OVI in a position to assist other countries with the serotyping of their viruses. In 1963 the World Organisation for Animal Health (OIE) designated the OVI as an OIE reference centre for BT, a function that it currently still performs [46].

The demonstration of the antigenic diversity of BTV field isolates combined with the successful adaptation and propagation of BTV in fertile hen’s eggs and later cell culture, led to the development of effective multivalent cross-protective vaccines that supplanted the old Theiler vaccine. In the mid 1940s a lyophilized chicken embryo-propagated quadrivalent attenuated vaccine had been developed, which was highly effective in protecting sheep against BT [53]. This vaccine was later replaced with 3 cell culture adapted pentavalent vaccines each consisting of 5 serotypes, that are still used to vaccinate sheep in southern Africa today [49].

The development of *in vivo* and *in vitro* systems for the large scale propagation of BTV in the 1940s paved the way for physiochemical, antigenic and genetic studies of the virus as well as the development of diagnostic assays. These studies indicated that BTV was a dsRNA virus that contained ten linear genome segments [2] enclosed within a triple layered protein capsid [54], eventually leading to its classification into a newly created *Orbivirus* genus [55]. The cloning of the genome segments of 21 southern African serotypes in the 1970s-1980s also led to the development of cross-hybridization assays which could be used to detect and type BTV field isolates. These studies identified segment 5 (encodes NS1) as the most appropriate segment for the diagnosis of BTV, whereas segment 2 (encodes VP2) was identified as being suitable for serotyping of field isolates [56].

### Global recognition of bluetongue

In the 1940s it was thought that BT was confined to southern Africa and that outbreaks of the disease in other regions of the world reflected the emergence of the virus from the African continent [18]. This assumption was soon challenged by the recognition of the disease in geographically widespread regions outside Africa. The first recognized outbreak of BT outside Africa occurred in Cyprus in 1943 [39]. Bluetongue virus was thereafter recognized as the causative agent of “sore muzzle” in sheep in California in 1952 [57] and in 1956–1957 a severe outbreak of BT that killed approximately 180 000 sheep occurred in the Iberian Peninsula [58]. It was initially thought that Australia was free from BT; however BTV-20 was isolated from *Culicoides* from the Northern Territory of the country in 1976 [59]. Bluetongue has subsequently been isolated from many regions in Africa, the Americas, the Middle East, the Indian subcontinent, China, south East Asia, northern Australia and Europe and it is thought that only Antarctica is currently free of BTV infection [23].

The large scale outbreak of BT in the Iberian Peninsula in the mid 1950s highlighted the threat which the disease could potentially pose to the supposedly BT free nations of Europe and Australia. The World Organisation for Animal Health (OIE) therefore classified BT as a list “A” disease in the mid 1960s. This decision for many years justified the imposition of strict import and export regulations on animals and their germ plasm from BT affected regions. Paradoxically the imposition of these regulatory measures had a more severe economic impact on BT affected countries and the global livestock industry than the disease itself [18]. With the recent spread of the disease into previously unaffected regions of northern Europe (including Scandinavia) and the incursion of several additional serotypes into south-eastern regions of the United States and northern Australia [21,60], BT has again risen in prominence as one of the important agricultural diseases of the 21^st^ century.

### Bluetongue in South Africa

Bluetongue is commonly seen in South Africa in late summer and autumn (between February to April), especially in areas with high rainfall and after good rains. In colder regions such as the southern Free State, the disease usually disappears with the occurrence of the first winter frosts, while it is probable that in warmer regions such as the KwaZulu Natal and Limpopo provinces the disease may be transmitted throughout the year. It has been suggested that the occurrence of BT in sheep in late summer and autumn in the country reflects the build-up of BTV in vector competent midge species during spring and early summer. A primary infection cycle may involve either wild antelope or cattle with sheep subsequently becoming infected as a result of “spill over” in a secondary infection cycle [38,61].

Bluetongue is a notifiable disease in terms of the South African Animal Diseases Act of 1984. Adherence to the act is however poor and outbreaks are not always reported. Only wet seasons with a large number of outbreaks serve to raise any level of concern. No routine surveillance for BT is carried out and information regarding serotype prevalence is based on the retrospective analysis of samples that are sporadically submitted to the OVI, as well as a limited number of field surveys. These surveys have indicated that 22 of the known 26 serotypes are present in South Africa, with serotypes 20, 21, 25 and 26 being considered to be exotic. An analysis of 258 ovine samples that were submitted to the OVI between 1983 and 2003 from all provinces of South Africa indicated that low denomination serotypes (1–4) were isolated most frequently and that some serotypes were extremely rare not having occurred for a period of more than 20 years (18, 19, 22, and 23). However, the analysis of these samples also indicated the presence of serotype 17, which had up till then not been detected in the country [62]. A single molecular epidemiological study using the NS3/A gene of BTV has been conducted in South Africa [63]. In this study different serotypes that were collected from widespread regions throughout the country were analyzed. The phylogeny that was inferred from the nucleotide sequence data of the NS3/A gene indicated that BTVs in South Africa cluster into two distinct lineages irrespective of their serotype, geographic area and year of isolation, suggesting that the transmission of these lineages is not restricted to any particular vector species or area of the country. Interestingly BTV isolates from the United States clustered into both lineages, supporting the notion that more than one introduction of BTV from southern Africa may have occurred into the United States in the past.

In South Africa multiple serotypes circulate each vector season however the occurrence of different serotypes is unpredictable and is most likely influenced by herd immunity. Monitoring of *Culicoides* spp. between 1978 and 1985 at various sites throughout South Africa indicated that 14 to 18 different serotypes were encountered every season, although at varying frequencies [64]. Usually three to five serotypes were isolated predominantly. These serotypes were replaced with other dominant serotypes the following season, just to become dominant again three to four years later. Serotypes which were most commonly encountered included 1–6, 8, 11 and 24. Serotypes 9, 10, 12, 13, 16 and 19 were encountered every season but at a much lower frequency, while serotypes 7, 15 and 18 were encountered only sporadically. Serotypes 1–6, 8, 11 and 24 are believed to have a high epidemic potential, whereas serotypes 1–6 and 10 which are more often associated with clinical disease in sheep are thought to have a high pathogenic index [38,49].

Bluetongue virus has been isolated from many different *Culicoides* species in South Africa, however only two species, *Culicoides imicola* and *Culicoides bolitinos,* are recognized as significant in the transmission of BTV. *Culicoides imicola,* whose larvae primarily inhabit organically enriched soils, is the dominant vector of BT in regions throughout South Africa [65]. *Culicoides bolitinos* is almost as widespread as *C. imicola*, but is dominant in the cooler regions of the country. This species, which breeds primarily in the dung of domestic and wild herbivores [66], has been shown to be significantly more susceptible to oral infection with BTV than *C. imicola*[65,67,68]. Bluetongue virus has also occasionally been isolated from mosquitoes that may play a role in the mechanical transmission of the virus [62].

The susceptibility of wild ruminants to BT in South Africa was first established by the experimental infection of blesbuck (*Damaliscus albifrons*). Blesbuck developed a sub-clinical infection with a sufficiently high virus titre to infect sheep that were experimentally injected with infected blood [69]. Evidence has subsequently been obtained that many wild African herbivore species are susceptible to infection, however these animals do not develop clinical disease. A survey was undertaken in 1997 in which wild African herbivores were sampled throughout South Africa from regions with differing vegetation and rainfall. Of the more than 18 species that were sampled, 10 tested positive for BTV antibodies. Species that had the highest sero-prevalences included the African elephant (*Loxodonta africana*), blue wildebeest (*Connochaetes taurinus*), black wildebeest (*Connochaetes gnou*) and buffalo (*Syncerus caffer*). Other species that tested positive included Red hartebeest (*Alcelaphus buselaphus*), impala (*Aepyceros malampus*), eland (*Taurotragus oryx*), blesbuck, gazelle (*Antidorcas marsupialis*) and giraffe (*Giraffa camelopardalis*) [70].

Following reports of deaths and abortions among pregnant bitches that were vaccinated with a BTV-11 contaminated commercial modified-live virus vaccine (canine distemper virus, canine parainfluenza virus, canine adenovirus-2 and canine parvovirus) [71], a structured survey of BTV sero-prevalence was conducted amongst wild and domestic carnivores in regions of southern and eastern Africa. This study indicated that a wide range of African carnivores can be infected with the virus. Species that tested seropositive included cheetah (*Acinonyx jubatus*), lions (*Panthera leo*), African wild dogs (*Lycaon pictus*), spotted hyenas (Crocuta crocuta), jackal (*Canis spp.*), large spotted genets (*Genetta tigrina*), domestic dogs *(Canis familiaris*) and cats (*Felis catus*). Antibodies against 12 different serotypes were identified during the survey, with serotypes 3, 8, 13 and 17 being associated most commonly with the surveyed carnivore species. Wild carnivores that had the highest sero-prevalences included large free- ranging species such as lion, spotted hyenas and African wild dogs, whereas smaller carnivores such as jackal and cheetah had lower sero-prevalences. It is assumed that infection of larger carnivores follow the ingestion of BT infected carcasses, whereas smaller carnivores that scavenge on partially decomposed carcasses have correspondingly lower sero-prevalences [72]. Many questions remain to be answered with regards to BT infections in African carnivore species. It is unknown for example what impact BT has on the population structure of endangered species such as cheetah and African wild dogs and whether infections of wild and domestic carnivores play any role in the onward transmission of BT to domestic ruminants.

The total population of sheep in South Africa is approximately 28 million that are distributed across all 9 provinces of the country. Most indigenous African sheep breeds do not show clinical signs of BTV infection and therefore the disease is not considered to be of major concern in many sheep rearing rural communities in the country. A large population of susceptible improved European wool and mutton breeds in which outbreaks of clinical disease are common, are however commercially farmed in South Africa [38,62]. From 1998 to 2000 the number of outbreaks reported per annum amongst sheep varied from 28 to almost 100, with the majority of outbreaks occurring during the wettest years [62]. Most unvaccinated sheep are infected with BTV at an early age and BTV antibody-negative sheep are therefore not readily found. For example, a sero-prevalence survey for BTV-specific antibodies was conducted amongst Merino sheep in the high-lying regions of the Eastern Cape, where sheep are not vaccinated since BT disease is not recognized. This survey indicated that a large proportion of Merino sheep that were bled in early spring and autumn tested positive for antibodies against the virus. Sero-prevalences amongst Merino sheep varied on a monthly basis from 1% to 84% [62]. A second survey was conducted in the wet, fairly tropical and low lying regions of KwaZulu Natal. In this survey a primarily unvaccinated rural/communal population of indigenous livestock or crosses thereof was targeted. In total 2852 animals were sampled of which a quarter consisted of sheep and the remainder consisted of goats. BTV antibodies were found in 63.7% of these animals [62].

Cattle typically do not develop clinical signs of BT and are rarely tested or vaccinated. In 1996 however, during an exceptionally wet year, BT was reported in cattle from regions throughout South Africa. Clinical signs included stomatitis, coronitis, lacrimation, salivation, nose and teat sloughs and haemorrhagic diarrhoea. The morbidity and mortality amongst infected animals were low, but a marked reduction in milk production was noted [73]. Twenty one BTV and nine epizootic haemorrhagic disease virus (EHDV) isolates were made from cattle during that season at the OVI, with co-infection with the two viruses apparently exacerbating the severity of clinical signs. The BTV isolates that were responsible for the outbreak were typed as serotypes 2, 3, 6 and 8, while the EHDV isolates were identified as serotype 6 [62].

### Diagnosis and control of bluetongue in southern Africa

Bluetongue is a well known disease in South Africa and a presumptive diagnosis is usually based on clinical signs. Several diseases and conditions may however be confused with BT in sheep. These include hepatogenous photosensitivity caused by plant and mycotoxin poisoning, foot-and-mouth disease, polyarthritis, foot rot, white muscle disease, heartwater and pulpy kidney disease. Clinical BT in cattle may be confused with malignant catarrhal fever, mucosal disease, foot-and-mouth disease, infectious bovine rhinotracheitis and epizootic haemorhagic disease [38]. Sheep suffering from bluetongue are rarely if ever euthanised. Farmers are advised to enclose affected sheep in small camps where food, water and shade are close at hand and the animals are left to recover on their own. Antimicrobial- and anti-inflammatory drugs are sometimes administered.

In South Africa the ubiquitous distribution of vector competent midge species and unvaccinated wild and domestic ruminants makes eradication of BT impossible. Control is therefore targeted to limit the economic impact of the disease amongst susceptible sheep. Practical procedures that limit the exposure of sheep to *Culicoides* bites include the avoidance of low-lying wet pasture, stabling of animals between dusk and dawn, shearing of sheep in early summer to allow for some wool growth before the high exposure months in late summer and autumn and the use of insect repellents [74]. The control of *Culicoides* in or around stables may also have some success in reducing the exposure of animals to *Culicoides* bites. The use of larvicides to kill larvae in soil and the drainage of water pools to reduce available breeding sites has also been suggested [75].

The most effective and cost effective method of protecting sheep against multiple circulating serotypes in South Africa remains the immunization of susceptible animals. The only vaccine that is widely used in southern Africa is produced by Onderstepoort Biological Products (OBP, Pretoria, South Africa) and consists of field strains that have been attenuated by serial passage in embryonated chicken eggs and BHK-21 cell culture. This vaccine is sold as 3 bottles, each containing 5 serotypes. Bottle A consists of serotype 1, 4, 6, 12 & 14; bottle B serotypes 3, 8, 9, 10 & 11 and bottle C serotypes 2, 5, 7, 13 & 19 [49]. Despite the fact that 22 serotypes of BTV have been isolated in southern Africa, only 15 serotypes are included in the current vaccine. Types 15, 16, 18 and 22–26 are not included due to their low pathogenicity for sheep, their low prevalence in *Culicoides*, or their relatively recent discovery [38].

The inclusion of specific serotypes in each vaccine bottle was done to prevent interference in the induction of immunity between the constituent strains due to serological cross-reactivity. The vaccine bottles are also formulated so that the most attenuated strains (slowest replicating strains) are applied first, thereby lessening the potential side effects of less attenuated strains (faster replicating strains) in the other vaccine preparations. As vaccine strains are able to replicate in the vaccinated host (albeit at a reduced rate) they are able to induce both humoral and cellular immune responses. The development of protective immunity is largely based on the development of serotype-specific neutralizing antibodies that may persist in vaccinated animals for several years. The vaccine also only demonstrates limited side effects in sheep in South Africa, which commonly includes a transient febrile reaction [49].

The individual vaccine bottles are usually applied to sheep at three week intervals, with immunity to most serotypes developing within three to four weeks after administration of the last vaccine bottle. Since immunity against all vaccine serotypes cannot be guaranteed, it is recommended that vaccination be repeated on an annual basis. In southern Africa, sheep are usually vaccinated with 1 ml of each of the three vaccines from August to October (spring). Due to the tendency of the cell culture adapted vaccine strains to cross the ruminant placenta, it is recommended that ewes be vaccinated 9–12 weeks before mating. Due to a transitory reduction in semen quality, rams should be vaccinated after the mating season. Furthermore the acquisition of colostral immunity necessitates that lambs be re-vaccinated at 6 months of age [49]. Although the OBP vaccine has not been registered for use in cattle, vaccine formulations containing BTV-2, 4, 9 and 16 have been used in cattle in several vaccination campaigns in southern Europe and the Middle East, with little adverse effects having been reported [76].

The vaccination of sheep in South Africa is not compulsory and on average only eight million doses of the vaccine is sold annually. The vaccine provides protection to approximately one third of the sheep in the country. A further one million doses is sold to neighbouring countries each year [49]. Various combinations of attenuated vaccine strains (3, 2, 4, 8–11, 16) that have been produced in South Africa, have also been used in the Iberian Peninsula, the eastern Mediterranean Islands, Italy, Spain, Portugal, France, Bulgaria and Israel [15,76,77].

Despite strict national and international standards with regards to manufacturing of the OBP vaccine, several disadvantages are associated with its use in the field. Documented side effects in sheep included severe to mild clinical signs [78], decreased milk production [76], transplacental infection [29-31], a transient reduction of semen quality in rams [79] and the suppression of non-specific lymphocyte blastogenesis when used in goats [80]. There is also experimental evidence that MLVs may have an increased potential for secretion in the semen of older bulls as well as rams [28]. In addition, attenuated vaccines are not DIVA compliant (able to distinguish between infected from vaccinated animals) which creates a problem when conducting surveillance for the disease [76]. Experimental studies have further demonstrated that MLVs are able to infect *Culicoides* and that the vaccine strains are able to replicate to a high enough titre in both the host and vector as to facilitate their onward transmission in the field [81,82]. Indeed several MLV strains that have been used in southern Europe and the Middle East have been isolated from *Culicoides* and sentinel animals. The persistence of MLVs in the field is of theoretical concern as these strains may possibly be able to reassort or recombine with other wild type or vaccine strains to yield novel viruses. These viruses may potentially display unique biological properties that may include enhanced virulence or an altered capacity to be transmitted by *Culicoides* in the field [14,15]. Furthermore, the prolonged circulation of MLV strains in the field may also potentially lead to a reversion to virulence as a result of the accumulation of point mutations (genetic drift) [81].

### Research on bluetongue in South Africa

The recent incursion of BTV into the previously unaffected regions of Europe has again stimulated interest in BTV research at the larger Onderstepoort complex. Cross-neutralization studies between the constituent strains of the live attenuated OBP vaccine in sheep are currently being conducted, in order to evaluate cross-protection between the viruses in the ruminant host. Serological cross-reactivity between different serotypes in the vaccine was originally evaluated in Guinea pigs, with little information being available on cross-reactivity in ruminant species. This work is being done with the eventual goal of reducing the number of serotypes that are included in the current OBP vaccine. There is also a particular interest in determining the immunological basis for differences in the clinical presentation of BT between different breeds of sheep, and on indentifying the genetic markers that influence the virulence and transmission potential of BTV. Several projects focusing on epidemiological questions with regards to the seasonal prevalence, overwintering and distribution of BT in South Africa are also in progress, while research on the pathogenesis of BTV in economically less important species such as goats, that may nevertheless play an important role in the epidemiology of BT, are also being conducted. Finally the endemicity of BTV in South Africa as well as the wide availability of vector competent midge species has made South Africa an attractive destination for international scientists that are interested in conducting *Culicoides* oral susceptibility studies.

## Conclusion

Due to the subclinical nature of BT in the majority of infected sheep, as well as the availability of effective attenuated vaccines, BT is thought to be of less importance today by veterinary authorities than other livestock diseases that are present in South Africa. Active surveillance for BT is not carried out and the economic impact which the disease has on the South African livestock industry is thus difficult to quantify. Nevertheless, not all farmers choose to vaccinate their animals and outbreaks of BT amongst unvaccinated sheep continue to occur on an annual basis. Sheep are exposed to a range of different serotypes in each vector season and several serotypes may be isolated from a single flock of sheep or an individual animal. Clinical disease amongst unvaccinated susceptible sheep in the field can be severe, and it is not uncommon for sheep farmers in South Africa to suffer significant losses amongst their flocks as a result of seasonal outbreaks of BT.

## Abbreviations

BT: Bluetongue; BTV: Bluetongue virus; MLVs: Modified-live virus vaccines; OVI: Onderstepoort Veterinary Institute; OIE: World Organisation for Animal Health; dsRNA: Double stranded ribonucleic acid; EHDV: Epizootic haemorrhagic disease virus; OBP: Onderstepoort Biological Products; DIVA: Distinguishing infected from non-infected.

## Competing interests

The authors declare that they have no competing interests.

## Authors’ contributions

PC was responsible for drafting the document, proof reading and manuscript preparation. MS, EHV, MM and MvV assisted with proof reading and manuscript preparation. The contents of this manuscript has been read and approved by all authors.

## Authors’ information

Mr. Peter Coetzee (MD) is a microbiologist at the Department of Veterinary Tropical Diseases at Onderstepoort, in Pretoria, South Africa. His interests include virology, molecular biology and the epidemiology of emerging animal pathogens.
